# Supplementary material for: The Central Domain of MCPH1 Controls Development of the Cerebral Cortex and Gonads in Mice
Source: Cells. 2022 Aug 31;11(17):2715. doi: 10.3390/cells11172715 (PMC9455054; doi:10.3390/cells11172715)
Supplement: Supplementary file 1 [file cells-11-02715-s001.zip › cells-1849957-supplementary.pdf]

# Supplementary Figures and Tables
**Wang *et al.***

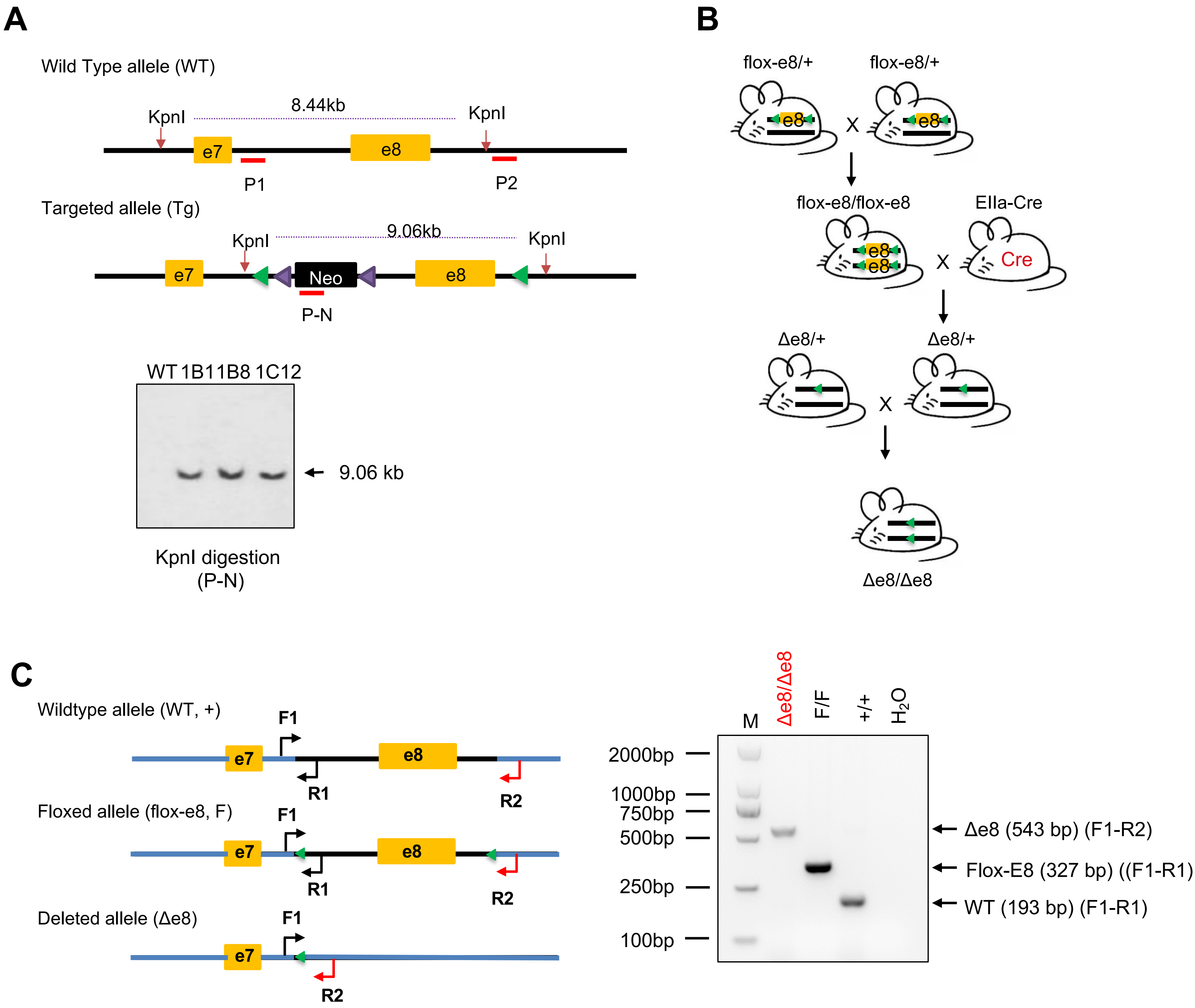


**Supplementary Fig S1**

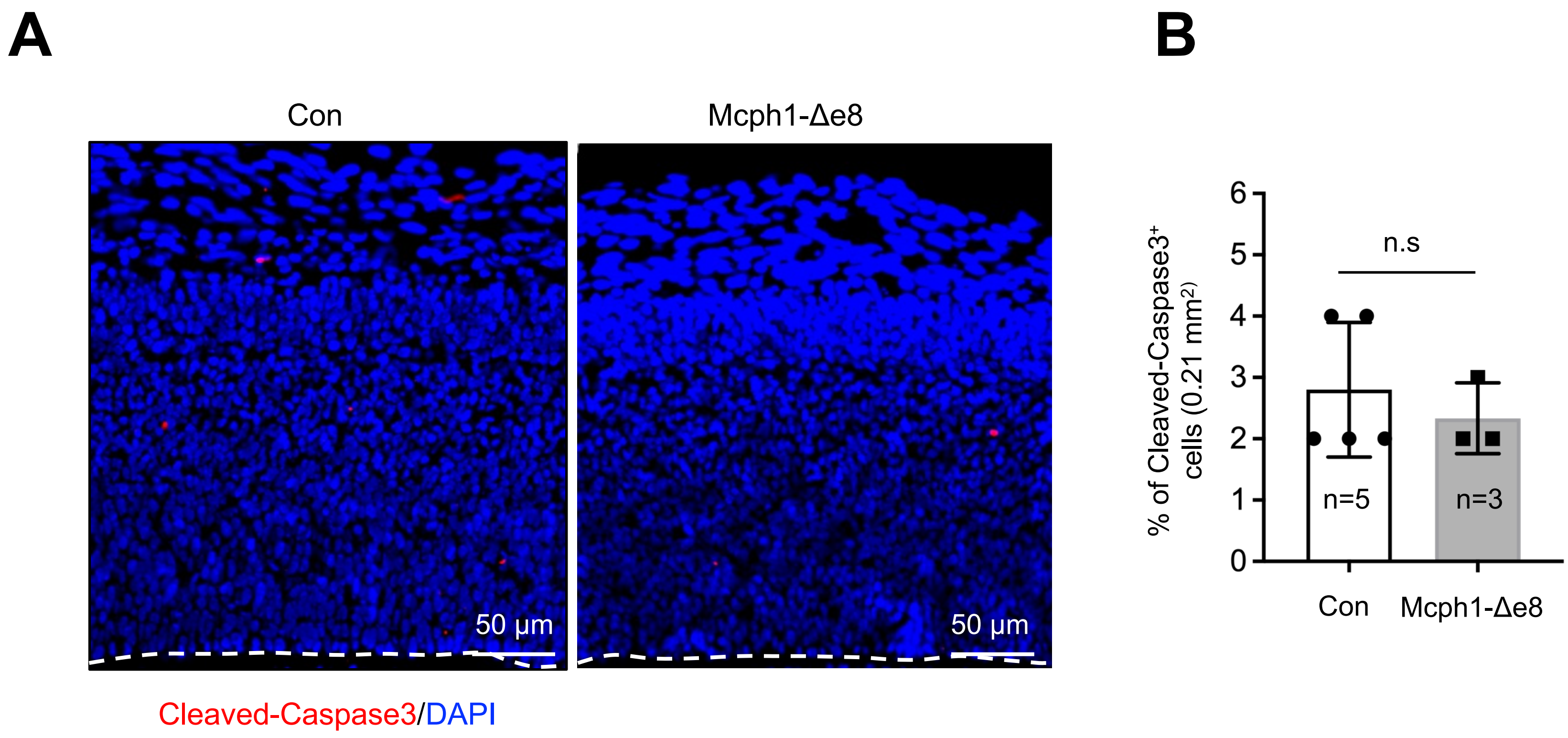


**Supplementary Fig S2**

Suppl Table S1: List of primers

| | |
|---|---|
| Mcph1-E8s | GTCCACAATCAGACTCATTC |
| Mcph1-E8as | CCTCAGGAGGAAGTCTCTCC |
| Mcph1-E7s | CCTCCCAGATGTTGGAGC |
| Mcph1-E9as | CTGCTGAGGTCTGACAAGGCC |
| Mcph1-E1s | ATGGAGGCCTCGGGAGGCGTT |
| Mcph1-E2as | CATATCCTCAAGTTGCTTTGC |
| Mcph1-E13s | TGAACTGGTACTCCTGTGTGGT |
| Mcph1-E14as | AGTTGGTAGTTGTTAAAGTCAC |

Suppl Table S2: List of primary antibodies

| Antigen | Species | Dilution | Application | Company |
|---|---|---|---|---|
| β-actin | Mouse | 1:10,000 | WB | Sigma-Aldrich |
| MCPH1 | Rabbit | 1:1,000 | WB | Cell Signaling |
| pS10 Histone3 | mouse | 1:400 | IF | Cell Signaling |
| Sox2 | Rabbit | 1:400 | IF | Abcam |
| Tbr2 | Rabbit | 1:400 | IF | Abcam |
| Ki67 | Rabbit | 1:400 | IF | Cell Signaling |
| Cleaved caspase-3 | Rabbit | 1:400 | IF | Cell Signaling |
| pS28 Histone3 | Rat | 1:400 | IF | Abcam |
| phospho Histone H2A.X (Ser139) | Mouse | 1:2,000 | WB | Sigma-Aldrich |
| phospho Chk1 (Ser317) | Rabbit | 1:1,000 | WB | Cell Signaling |

Suppl Table S3: List of secondary antibodies

| Antigen | Species | Conjugate | Dilution | Company |
|---|---|---|---|---|
| Mouse IgG | Goat | HRP | 1:2,000 | Proteintech |
| Rabbit IgG | Goat | HRP | 1:2,000 | Proteintech |
| Mouse IgG | Goat | Cy3 | 1:1,000 | Abcam |
| Rabbit IgG | Goat | Cy3 | 1:1,000 | Abcam |
| Rat IgG | Goat | Cy3 | 1:1,000 | Abcam |
